# Mining TCGA database for genes of prognostic value in gastric cancer microenvironment

**DOI:** 10.1111/jcmm.15595

**Published:** 2020-08-20

**Authors:** Qingzhi Lan, Peng Wang, Shan Tian, Weiguo Dong

**Affiliations:** ^1^ Department of Gastroenterology Renmin Hospital of Wuhan University Wuhan China; ^2^ Central Laboratory of Renmin Hospital Wuhan China; ^3^ College of Life Sciences Wuhan University Wuhan China

**Keywords:** estimate, gastric cancer, prognostic marker, tumour microenvironment

## Abstract

Gastric cancer (GC) is the sixth most common malignancy and the third leading cause of cancer‐related death worldwide. Emerging evidence suggests that tumour microenvironment cells play a vital role in the development and prognosis of GC. To investigate the possible effect of stromal scores and immune scores on the overall survival (OS) on the GC patients, we divided GC patients into ‘high’ and ‘low’ groups based on their stromal and immune scores, and found differentially expressed genes related to prognosis of GC patients. Functional enrichment analysis and GSVA further revealed that focal adhesion and ECM‐receptor interaction are associated with GC patients' survival. Finally, we analysed the effects of genes commonly involved in focal adhesion and ECM‐receptor interaction on GC patients' survival and validated our results in another GC cohort from GEO data sets. In conclusion, we obtained a list of tumour microenvironment‐related genes that predict poor prognosis in GC patients.

## 1 INTRODUCTION

Gastric cancer (GC) is the sixth most common malignancy and the third leading cause of cancer‐related death worldwide.^1^ Despite the declining morbidity as well as mortality and the significant advances in the comprehension of aetiology and molecular mechanisms, the burden remains high in Asia, Latin America, and eastern and central part of Europe.^2^ Although several treatment approaches are applied including surgery, chemotherapy, radiation therapy and molecular targeted therapies, the long‐term outcome of GC patients at advanced stages remains disappointing.^3^, ^4^

The tumour microenvironment (TME) refers to the environment in which cancer cells originate and develop. Except for cancer cells, the TME consists of different cell types (stromal cells, immune cells, endothelial cells, etc) and extracellular elements (chemokine, cytokines, hormones, etc).^5^, ^6^ Emerging evidence suggests that TME cells (including macrophages, T cells and fibroblasts) all play a vital role in the initiation and progression of GC.^7^, ^8^, ^9^, ^10^, ^11^ As two major cell types apart from cancer cells in the TME, stromal cells and immune cells exhibit important role in diagnostic and prognostic evaluation of solid tumours. Stromal cells can receive signals sent by cancer cells and then supply the cancer cells with a variety of growth factors, which are essential for invasive growth and metastasis.^12^, ^13^, ^14^, ^15^, ^16^, ^17^ On the other hand, the immune cells in the TME function in a context‐dependent way: tumour‐antagonizing effects of T cells in ovarian cancer^18^, ^19^, ^20^ and tumour‐promoting effects in colorectal cancer.^21^, ^22^ Hence, an overall understanding of stromal cells and immune cells may provide important vision into tumour biology and contribute to the development of reliable prognostic predictive models. Yoshihara et al^23^ designed an algorithm that used the unique properties of the transcription spectra of cancer samples to infer the number of tumour cells and infiltrating normal cells, called ESTIMATE (Estimation of STromal and Immune cells in MAlignant Tumour tissues using Expression data). Using this algorithm, researchers can calculate stromal scores and immune scores to predict the number of infiltrating stromal and immune cells. Subsequent reports quickly applied the ESTIMATE algorithms to prostate, breast and colon cancers, demonstrating the effectiveness of the big data–based algorithm.^24^, ^25^, ^26^ In this current study, we initially measured stromal scores and immune scores of GC patients from The Cancer Genome Atlas (TCGA, https://cancergenome.nih.gov) database by using ESTIMATE algorithm. Then, we explored the correlation between stromal scores and prognosis as well as clinical stages. Furthermore, we conducted GO and KEGG analysis by using differentially expressed genes between high and low stromal score groups. Finally, we validated our conclusion with GEO database. This research aims to develop new prognostic predictive biomarkers for GC.

## 2 MATERIALS AND METHODS

### 2.1 Database

**FIGURE 1:**
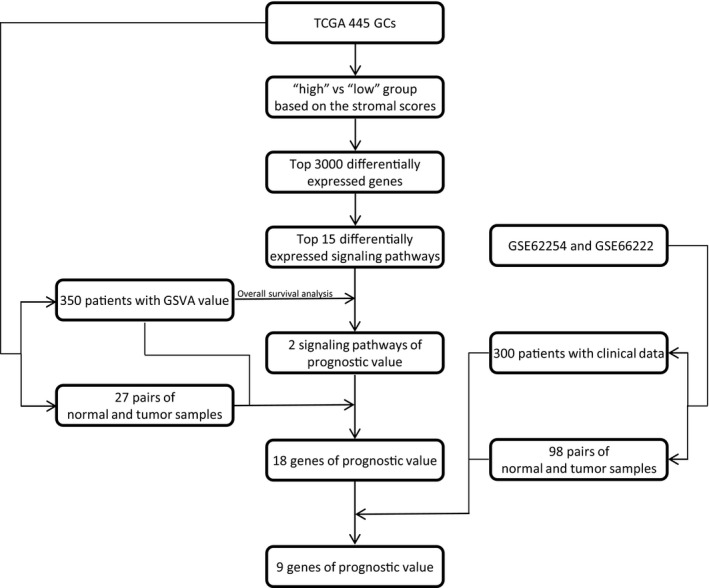
The flow diagram of this study

Figure 1 shows the flow diagram of our study. Gene expression profiles for STAD and copy number of the gene level were obtained from the UCSC Xena (https://xenabrowser.net). Clinical data such as gender, age, histological type and survival data were also downloaded from TCGA data portal (https://portal.gdc.cancer.gov/). Immune scores and stromal scores were calculated by applying the ESTIMATE algorithm to the downloaded gene expression profile using the R package ESTIMATE. Based on this, we obtained 375 samples with respective estimate values. In addition, we also downloaded ESTIMATE results from MD Anderson (https://bioinformatics.mdanderson.org/estimate/). So, we obtained 415 samples with respective estimate values in total.

### 2.2 Identification of differentially expressed genes (DEGs)

Data analysis was performed using package limma.^27^ Adj. *P*‐value < .05 and the 3000 most significant were set as the cut‐offs to screen for differentially expressed genes (DEGs).

### 2.3 Overall survival analysis

Kaplan‐Meier plots were generated to illustrate the relationship between patients' OS and gene expression levels of DEGs. The association was tested by log‐rank test.

### 2.4 Enrichment analysis of DEGs

Differentially expressed genes were used to perform GO analysis, including biological process (BP), molecular function (MF) and cellular component (CC) using R package clusterProfiler.^28^ Function enrichment analysis of DEGs was performed by the Database for Annotation, Visualization and Intergrated Discovery (DAVID), and the DAVID database was searched to perform pathway enrichment analysis with reference from Kyoto Encyclopedia of Genes and Genomes (KEGG) pathways. False discovery rate (FDR) <0.05 was used as the cut‐off, and the whole expression profiles were used to conduct GSEA using package GSEA.

### 2.5 Public data validation

Data were downloaded from GEO profiles (https://www.ncbi.nlm.nih.gov/geo/). Data processing and data analyses were completed through R software, as mentioned above.

## 3 RESULTS

### 3.1 Stromal scores are associated with GC stages and their OS

Firstly, we obtained gene expression profiles and clinical characteristics of all 443 GC patients initial pathologically diagnosed between 1996 and 2013 from TCGA database. Secondly, according to the ESTIMATE algorithm, we calculated the stromal scores and immune scores of 415 GC patients based on their respective RNA expression profiles. Among which, the ranges of stromal scores and immune scores were −1957.19 ~ 2085.81 and −1568.74 ~ 2826.73, respectively. The means of stromal scores and immune scores were 40.79993 and 613.2823, respectively.

**FIGURE 2:**
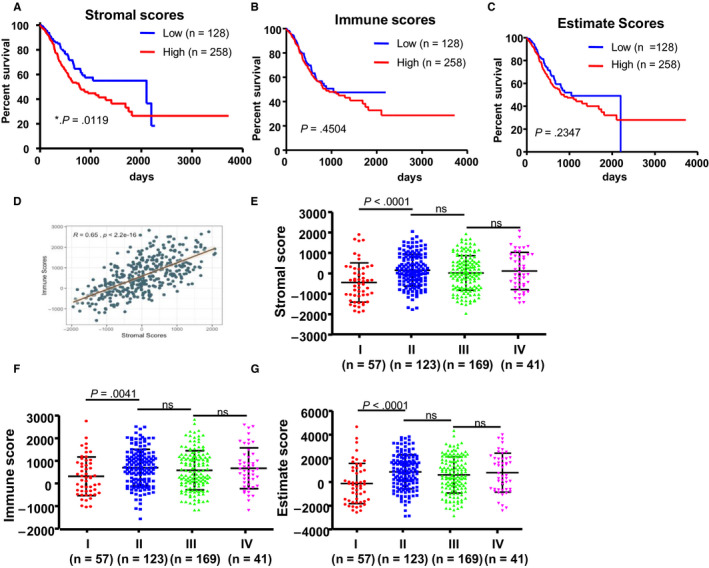
Stromal scores are associated with GC stages and their overall survival. A, STAD cases were divided into two groups based on their stromal scores: the top 2/3 of 258 cases with higher stromal scores and the bottom 1/3 of 128 cases with lower stromal scores. As shown in the Kaplan‐Meier survival curve, median survival of the low score group is longer than the high score group (2100 d vs 782 d), as indicated by the log‐rank test; *P*‐value is .0119. B, Similarly, STAD cases were divided into two groups based on their immune scores: the 2/3 of 258 cases and the 1/3 half of 128 cases. The median survival of the low score group is longer than the high score group (1043 d vs 869 d); however, it is not statistically different as indicated by the log‐rank test; *P* = .4504. C, Similarly, STAD cases were divided into two groups based on their estimate scores: the 2/3 of 258 cases and the 1/3 half of 128 cases. The median survival of the low score group is longer than the high score group (1043 d vs 869 d); however, it is not statistically different as indicated by the log‐rank test; *P* = .1688. D, Correlation analysis of stromal scores and immune scores. E‐G, Distribution of stromal scores, immune scores and estimate scores in the four different GC stages. Dot‐plot shows that there is a significant association between GC stages and the level of stromal scores, immune scores and estimate scores, respectively (n = 406, *P* < .001)

To investigate the possible effect of stromal scores and immune scores on the OS of the GC patients, we divided 386 GC patients with available survival profiles into ‘high’ and ‘low’ groups based on their stromal and immune scores. The top 2/3 of 258 cases were classified into ‘high’ group, and the bottom 1/3 of 128 cases were classified into ‘low’ group. As shown in the Kaplan‐Meier survival curves, median survival time of the low stromal score group was significantly longer than that of the high stromal score group (2100 days vs 782 days, *P* = .00119, Figure 2A). Similarly, patients with low immune scores also exhibited longer median OS time than that of patients with high immune scores (1043 vs 869, *P* = .4504, Figure 2B), although this difference was not statistically significant. To explore the overall effect of the TME on the GC patients, we added the stromal scores and immune scores to get estimate scores and divided patients into the high group and the low group as mentioned above. Results show that the median OS time of the low estimate score group was also longer than that of high estimate score group (1043 days vs 869 days Figure 2C), although it is not statistically significant (*P* = .1688). We noticed that the stromal scores and immune scores had similar effects on the OS of GC patients, and we wonder whether they correlated with each other. To validate our hypothesis, we analysed the correlation between immune scores and stromal scores, using Pearson's method. Results show that stromal scores and immune scores displayed strong correlations between each other (Figure 2D).

Due to lack of information of tumour stages of 25 cases, we obtained 390 out of 415 cases which have the respective tumour stage diagnoses. Among the 390 patients, tumour stage diagnoses included 57 (14.6%) cases of stage I, 123 (31.5%) cases of stage II, 169 (43.3%) cases of stage III and 41 (10.5%) cases of stage IV. The average stromal scores of stage II cases ranked the highest of all four stages, followed by that of stage IV, and stage III. The stage I cases possessed the lowest stromal scores (Figure 2E, *P* < .0001). Similarly, the rank order of immune scores and estimate scores of GC stages was stage II > stage IV > stage III > stage I (Figure 2F,G, *P* = .0041, *P* < .0001, respectively).

With the aim to identify more specific targeted molecule for GC therapy, we screened all of the genetic mutations in the GC genome based on TCGA data sets and picked out seven most common mutational genes, which included MYC, MET, KRAS, MST1, NRAS, HRAS and TP53. As is shown in Figure S1A, MYC was the most common mutant gene in the whole 411 GC patients. We plotted the distribution of stromal scores based on the status of MYC mutation in GC patients and results show that MYC‐mutant patients had lower stromal scores (*P* = .0904, Figure S1B). Survival analysis shows that patients in the MYC‐mutant group exhibited shorter OS than that of patients in the MYC‐wild‐type group (*P* = .0663, Figure S1C). We wonder whether MYC‐mutant and high stromal scores have superimposed effects on the survival of GC patients. To verify this, we combined the status of MYC gene and the stromal scores, and then, we divided 411 patients into four groups and analysed their survival. The results demonstrated the MYC‐mutant plus high stromal scores have the worst prognosis and the MYC‐wild‐type plus low stromal scores possess the longest median survival. We also noticed in the MYC‐wild‐type condition, patients with high stromal scores exhibited a longer median survival than low stromal scores. Taken these results together, we can conclude MYC‐mutant and high stromal scores have superimposed effects on the survival of GC patients (Figure S1D).

### 3.2 Comparison of gene expression profile with stromal scores in GC

**FIGURE 3:**
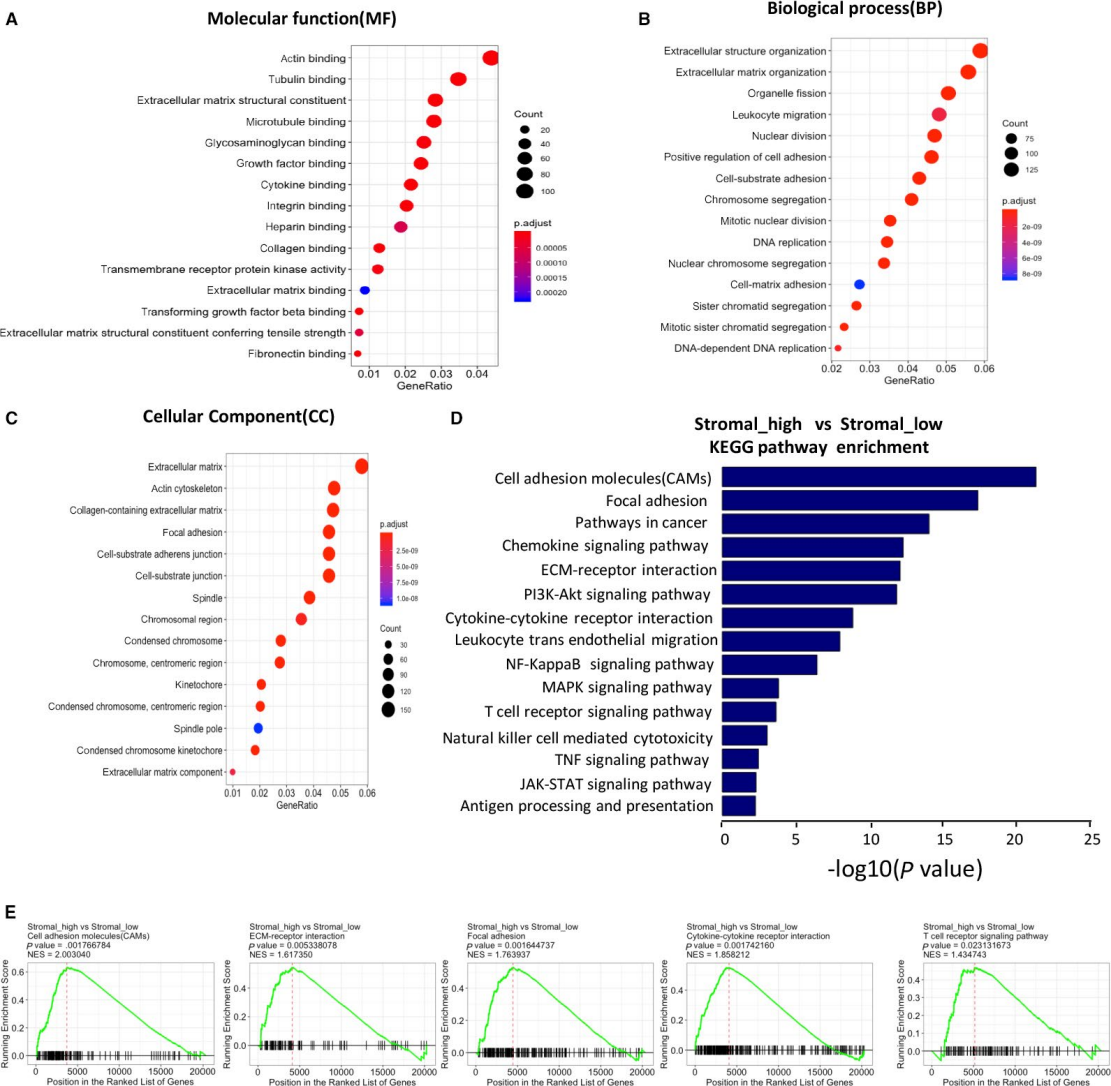
Comparison of gene expression profile with stromal scores in GC. A‐C, GO analysis to explore the 3000 most different genes participate in molecular function (MF) (A), biological process (BP) (B) and cellular component (CC) (C). D, To explore the 3000 most different genes involved in signalling according to the Kyoto Encyclopedia of Genes and Genomes (KEGG) data sets. E, GSEA of the data of RNA‐sequencing, which show these KEGG signalling pathways are up‐regulated in the ‘stromal_high’ group

To explore why patients with high stromal scores showed poor clinical outcome, we took advantage of gene expression profiles of GC patients in TCGA data set. We selected the top 60 cases as the ‘high’ group and the bottom 30 cases as the ‘low’ group. Then, we compared the RNA‐sequence data of the ‘high’ and ‘low’ groups using the Limma (R packages) to obtain the matrix of differentially expressed genes. We selected top 3000 genes to perform downstream analysis. To identify the probable function of the 3000 most differentially expressed genes between high and low stromal score groups, we performed gene ontology analysis using the R package ‘clusterProfiler’, and top 15 GO terms in molecular function (MF) (Figure 3A), biological process (BP) (Figure 3B) and cellular component (CC) (Figure 3C) are shown. Top GO terms included extracellular matrix structure constituent, collagen binding, extracellular matrix binding, extracellular matrix organization, focal adhesion and cell‐substrate junction. The results obtained from MF, BP and CC confirmed these genes participate in the biological process in the extracellular matrix (ECM). In addition, we performed Kyoto Encyclopedia of Genes and Genomes (KEGG) signalling pathway enrichment analysis and found some signalling pathways participating in extracellular matrix such as cell adhesion molecules, focal adhesion, ECM‐receptor interaction and some participating in immune and inflammatory responses, such as chemokine signalling pathway, cytokine‐cytokine receptor interaction, T cell receptor signalling pathway and antigen processing and presentation (Figure 3D).

In order to explore whether the signalling pathway obtained from GO and KEGG signalling pathway enrichment analysis was up‐regulated or down‐regulated, we performed gene set enrichment analysis (GSEA) using the whole matrix of differentially expressed genes, which showed these signalling pathways involved in extracellular matrix, immune and inflammatory response, and chemokine activities and integrin binding were up‐regulated in high stromal score group (Figure 3E, Figure S2A).

### 3.3 Correlation of expression of individual signal pathway in overall survival in TCGA

**FIGURE 4:**
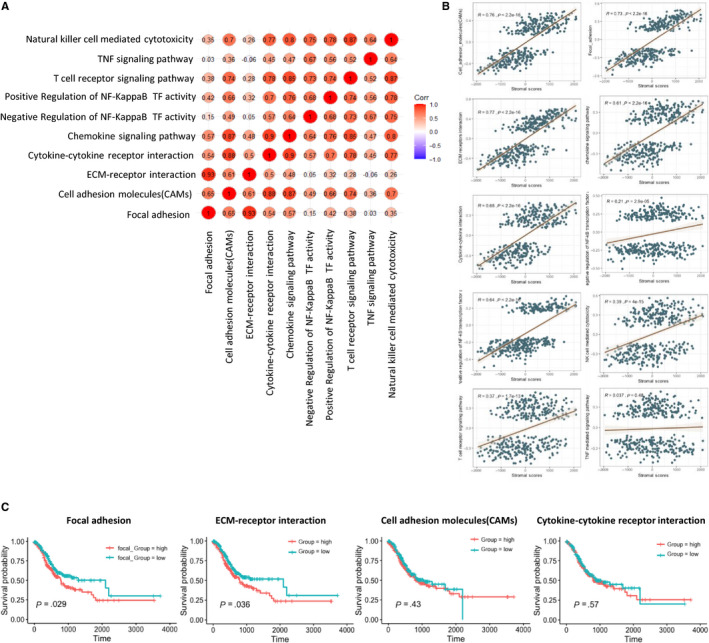
Correlation of expression of individual signal pathway in overall survival in TCGA. A, Correlation of the GSVA value of the 10 signalling pathways, which may take part in the poor clinical performance of the ‘stromal‐high’ group. B, Correlation between stromal scores and the GSVA values of the 10 signalling pathways, most of which are positive correlation, apart from TNF‐mediated signalling pathway. C, Survival analysis was performed on N = 350 patients obtained from the TCGA cohort of gastric cancer patients that had long‐term clinical follow‐up data. Displayed gene sets are downloaded from http://www.gsea‐msigdb.org, most of which are downloaded from KEGG and GO data sets; GSVA scores of each signalling pathway are performed using R package GSVA; for each signalling pathway, the top 1/2 of 175 cases with higher GSVA scores are ‘high’ group, and the bottom ½ of 175 cases with lower stromal scores are ‘low’ group

Our data show the high stromal scores were linked to the up‐regulation of signalling pathway involved in extracellular matrix, immune and inflammatory response, and chemokine activities and integrin binding. To further reveal whether they have correlation between each other, we selected 10 signalling pathways generated from KEGG pathway enrichment analysis, which are shown in Figure 4A. We downloaded gene sets of each signalling pathway from GSEA MSigDB (https://www.gsea‐msigdb.org/gsea/msigdb). Based on the gene sets and the TCGA gene expression profiles, we calculated GSVA value of each signalling pathway using the R package GSVA. According to the GSVA values, we obtained the correlation coefficients between the 10 signalling pathways. We observed most of them have a correlation between each other, especially between ECM‐receptor interaction and focal adhesion, cytokine‐cytokine receptor interaction and cell adhesion molecules, chemokine signalling pathway and cell adhesion molecules, chemokine signalling pathway and cytokine‐cytokine receptor interaction, natural killer cell‐mediated cytotoxicity and T cell receptor signalling pathway (Figure 4A). In addition, we evaluated the correlation between the ten signalling pathways and stromal scores and find that most of them are positively related to stromal scores, excepted for TNF‐mediated signalling pathway (Figure 4B). Furthermore, we conducted survival analysis of 350 patients with complete clinical information and learned that median OS of patients in the low GSVA value of focal adhesion and ECM‐receptor interaction group is significantly longer than that in the high GSVA value group (Figure 4C), whereas the remaining eight signalling pathways have little effect on GC patients' survival (Figure S2B). So, we can conclude that up‐regulated focal adhesion and ECM‐receptor interaction may promote the stromal cells to infiltrate into TME, and predict poor prognosis in GC patients.

### 3.4 Correlation of expression of individual DEGs in overall survival in TCGA

**FIGURE 5:**
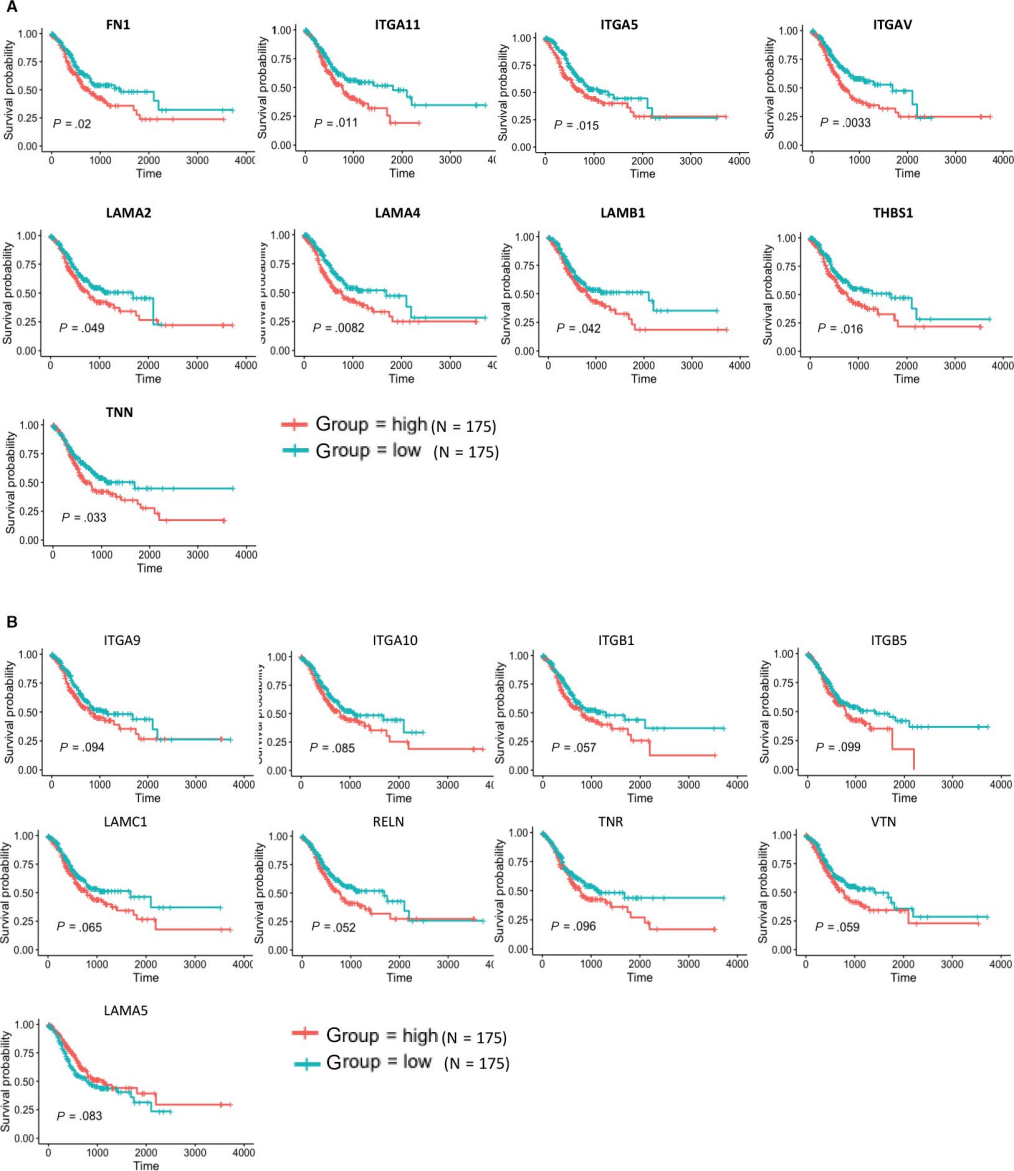
Correlation of expression of individual DEGs in overall survival in TCGA. Kaplan‐Meier survival curves were generated for selected DEGs extracted from the comparison of groups of high (red line) and low (blue line) gene expression. *P* < .05(A) or *P* < .1(B) in log‐rank test. OS, overall survival in days

**FIGURE 6:**
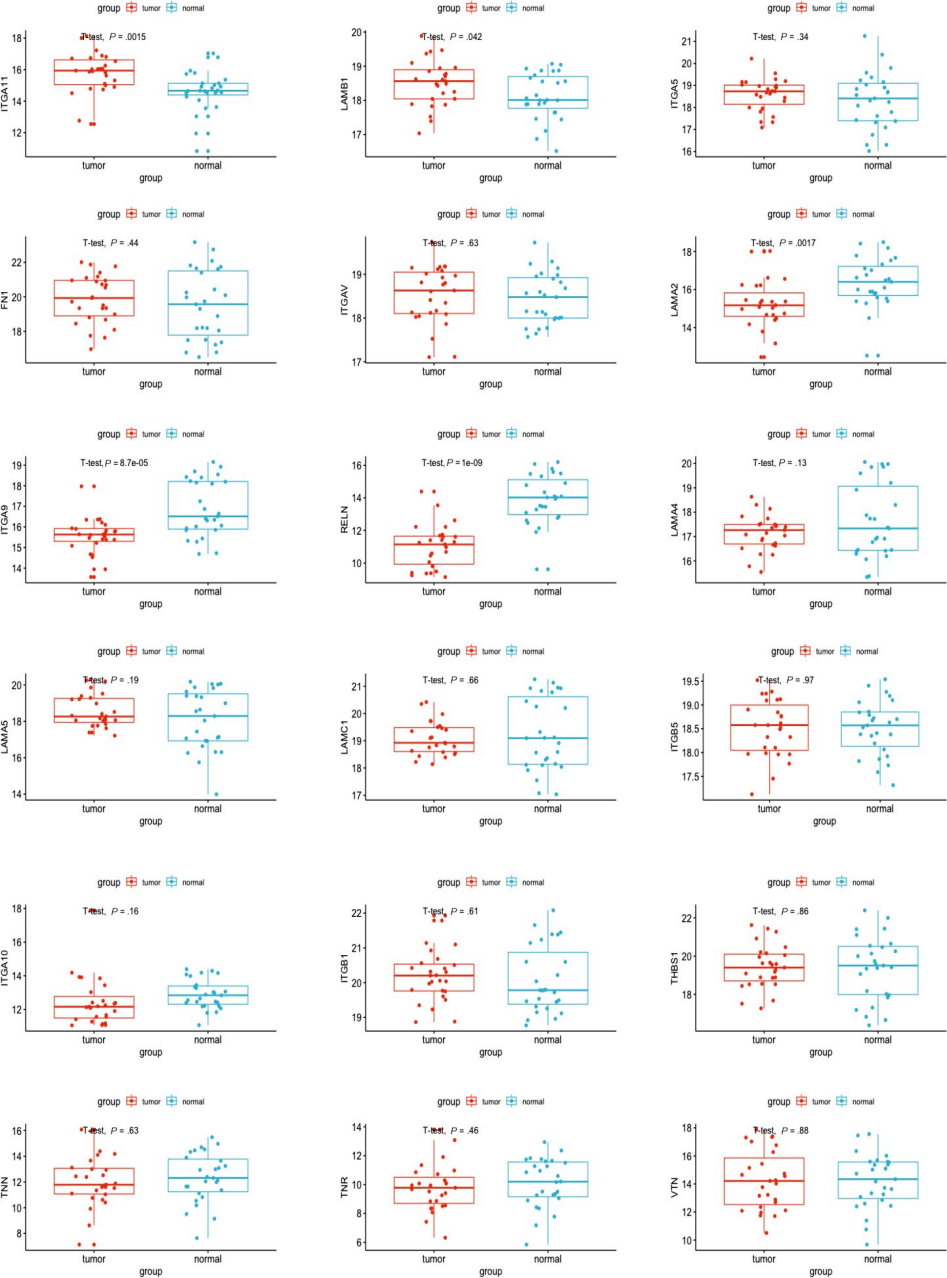
Different expression levels of the marker genes in TCGA cohort. Expression levels of 18 genes in Figure 4 in 27 pairs of tumorous samples and patient‐matched normal samples in TCGA cohort

As only focal adhesion and ECM‐receptor interaction are associated with GC patients' survival, we selected 63 genes commonly involved in focal adhesion and ECM‐receptor interaction. Results indicate that 9 of 63 genes are statistically associated with GC patients’ survival such as FN1, ITGA11, ITGA5, ITGAV, LAMA2, LAMA4, LAMB1, THBS1 and TNN (*P* < .05) (Figure 5A), whose higher expression level may predict poor prognosis, whereas 9 of 63 genes have the same effects on the GC patients' survival, although not statistically significant, such as ITGA10, ITGA9, ITGB1, ITGB5, LAMA5, LAMC1, RELN, TNR and VTN (*P* < .1) (Figure 5B). With the aim of finding novel biomarkers for GC diagnosis and prognosis assessment, we compared expression levels of these genes in 27 pairs of tumorous tissues and patient‐matched normal tissues and found only 2 of 18 genes (ITGA11 and LAMB1) expressed significantly higher in tumorous tissues than normal tissues (*P* < .05) (Figure 6), whereas 3 of 18 genes (LAMA2, ITGA9 and RELN) expressed significantly lower in tumorous tissues (*P* < .05) (Figure 6).

### 3.5 Validation of correlation of DEGs extracted from TCGA database in public data sets

**FIGURE 7:**
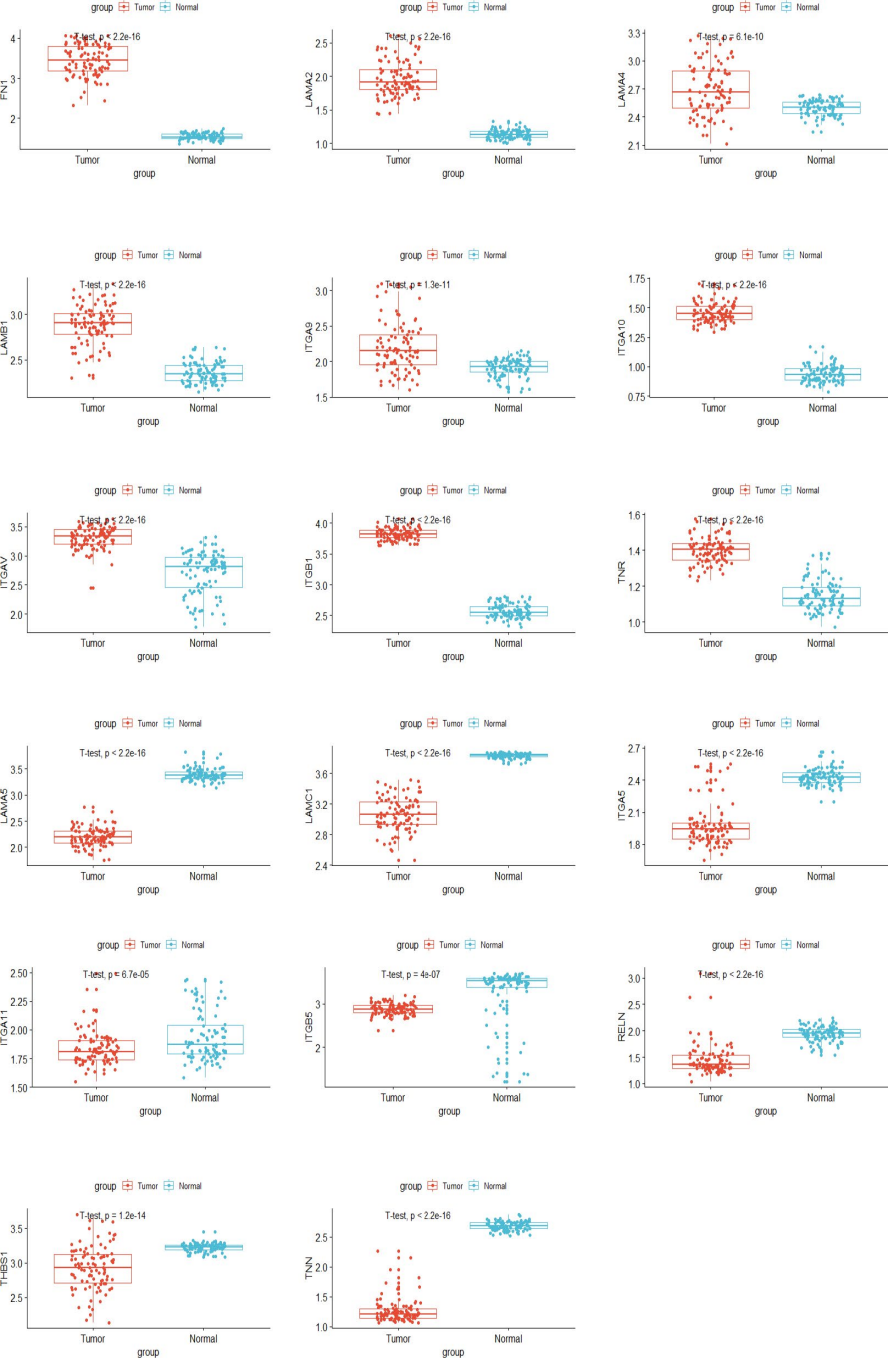
Validation of different expression levels of the marker genes in public data sets. Expression levels of 17 genes in 98 pairs of tumorous samples and patient‐matched normal samples in GEO cohort

**FIGURE 8:**
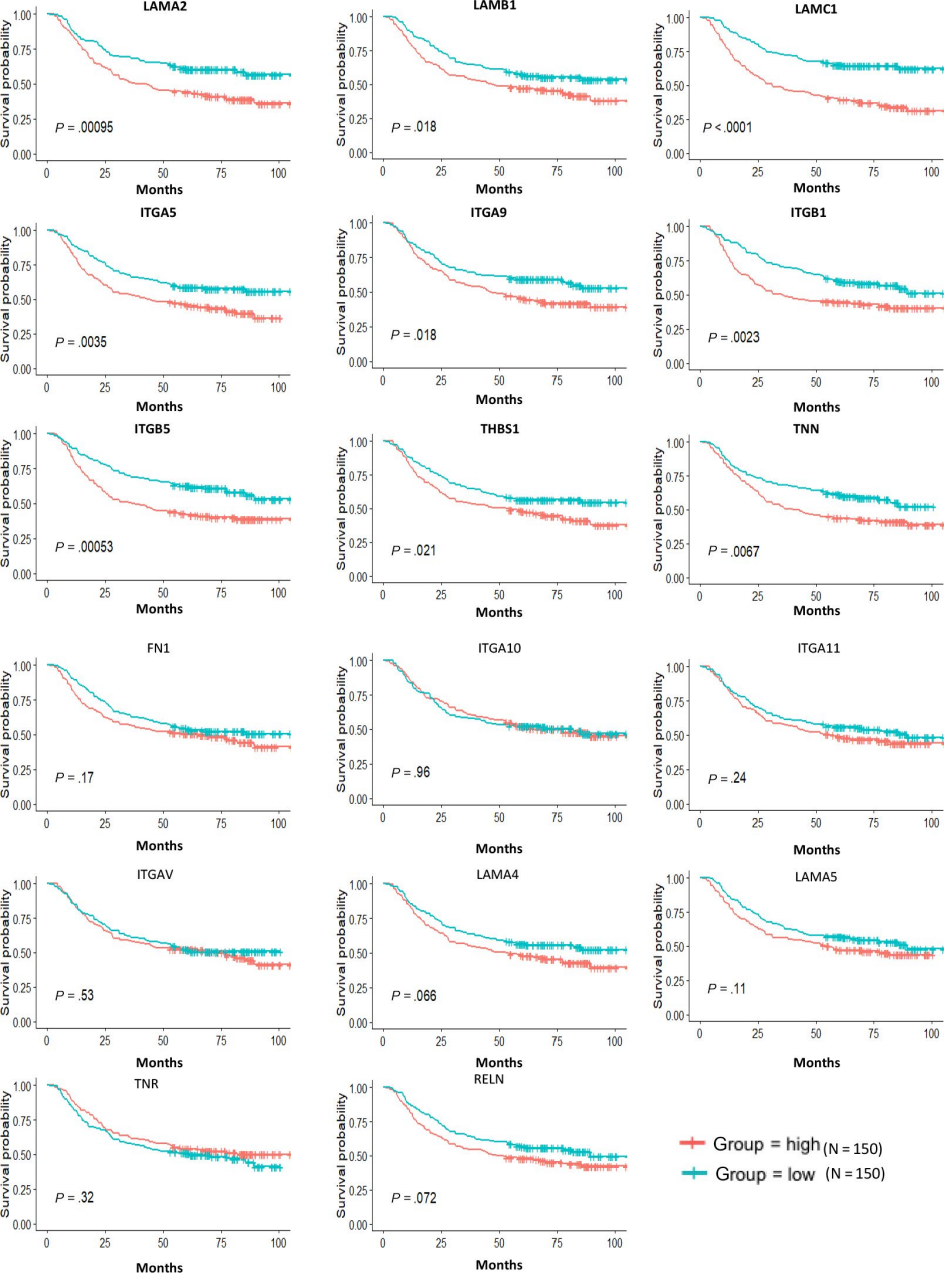
Validation of correlation of DEGs extracted from TCGA database with overall survival in public data sets. Kaplan‐Meier survival curves were generated for selected DEGs extracted from the comparison of groups of high (red line) and low (blue line) gene expression

To confirm our conclusion, we downloaded public data sets from GEO profiles (https://www.ncbi.nlm.nih.gov/geo/), and we noticed a series of studies conducted by the ACRG (Asian Cancer Research Group), GSE62254 and GSE66222. GSE62254 was composed of microarray profiles from 300 GC patients. Firstly, we compared the expression levels of these 17 genes in 98 pairs of tumorous tissues and patient‐matched normal tissues. As is shown in Figure 7, expression level of LAMB1, FN1, ITGAV, LAMA2, LAMA4, TNR, ITGB1, ITGA10 and ITGA9 is higher in tumorous tissues than that of patient‐matched normal tissues (*P* < .05), whereas other eight genes, such as ITGA11, ITGA5, THBS1, TNN, LAMA5, ITGB5, RELN and LAMC1, express lower in tumorous tissues than patient‐matched normal tissues (*P* < .05). Apart from this, we also performed the survival analysis based on the 300 tumorous samples with patient‐matched clinical data. As is shown in Figure 8, the effects of these genes on patients' survival are consistent with that from TCGA. Patients with high expression level of these genes exhibited shorter median OS than that of patients with lower expression level. These results further confirm the conclusion we obtained from TCGA cohort.

## 4 DISCUSSION

In this study, we aimed to identify TME‐related genes that were related to OS of GC. By taking advantage of TCGA database and ESTIMATE algorithm, we found that GC patients with low stromal scores exhibited longer median OS than that of patients with high stromal scores. In addition, we conducted GO and KEGG analysis by using top 3000 differentially expressed genes between high and low stromal score groups and results show that 18 genes related to focal adhesion and ECM‐receptor interaction are associated with GC patients' survival. Moreover, by cross‐validation with GEO data sets, we identified nine tumour microenvironment‐related genes to be significantly associated with poor prognosis of GC patients, which were ITGA5, LAMA2, LAMB1, THBS1, TNN, ITGA9, ITGB1, ITGB5 and LAMC1.

Of these 18 genes obtained from TCGA, four genes (FN1,^29^, ^30^ LAMA4,^31^ RELN^32^ and ITGB1^33^, ^34^) have been reported to be involved in GC pathogenesis or related to patient prognosis. The remaining 14 genes have not been reported to be involved in the prognosis of GC and could be used as potential biomarker. These include integrin family genes ITGA5, ITGA9, ITGA11, ITGAV and ITGB5; laminin family genes LAMA2, LAMA5, LAMB1 and LAMC1; cell adhesion molecules THBS1 and VTN; and extracellular matrix glycoproteins TNR and TNN. Of these nine genes we validated in GEO data sets, we are especially interested in LAMB1 and ITGA5. ITGA5 is a member of integrin family, which mediates the communications between different cells or between cells, and extracellular matrix (ECM) has been reported to be correlated with the progression of colorectal cancer,^35^, ^36^ pancreatic cancer,^37^ hepatocellular carcinoma,^38^ oral squamous carcinoma,^39^ non‐small‐cell lung cancer^40^ and bladder cancer.^41^ LAMB1 encodes laminin β‐1, a member of extracellular matrix glycoproteins, which is the major noncollagenous constituent of basement membranes, and has been involved in a wide variety of biological processes including cell adhesion, differentiation, migration and metastasis. It has been reported to be significantly higher in the serum of CRC patients and had a better diagnostic performance compared to CEA.^42^

Previous studies show that components in TME play a vital role in the initiation and progression of GC. Tumour‐associated macrophages, as the major component of TME, can produce exosomes to enhance cytoskeleton‐supporting migration of GC both in vitro and vivo by activating PI3K‐Akt signalling pathway.^43^ Another component of TME, mesenchymal stem cells (MSCs), is found to derive exosomes that enhance GC malignant properties and induce the epithelial‐mesenchymal transition (EMT) and cancer stemness in GC cells by activating the Akt signalling pathway.^44^, ^45^, ^46^ Cancer‐associated fibroblasts, differentiated from MSCs, have also been reported to show potential effects on various GC models, including carcinogenesis, metastasis, invasion, angiogenesis, resistance to therapy and tumour immunity.^4^ In the current research, we found that GC patients with high stromal score or high immune score or high expression of TME‐related genes exhibited shorter OS, which is consistent with previous results that TME promotes carcinogenesis, invasion and metastasis of GC. Although some of our results did not show statistical significance, we assumed that the sample size of our study is not big enough and needs further research.

Our study has strengths and limitations. Thanks to the rapid development of whole‐genome sequencing and establishing of public database, researchers like us can freely access to these resources and conduct big data analysis of large GC cohorts. Based on the resources obtained from public database, we extracted and validated a list of TME‐related genes, which may affect the development of GC and overall survival of patients. However, we noticed that some genes have a distinct expression pattern between TCGA cohort and GEO data sets. We speculate that it may be because (a) the sample size in TCGA is too small, (b) they adopted different transcriptome technologies and data processing methods, and (c) their standards for surgical sampling were inconsistent. In addition, our study is limited to bioinformatics analysis and no additional experiments are performed to verify our conclusions. Hence, our future work will focus on the functions of these genes on GC via experimental researches in GC cell lines and patients.

In brief, by making use of TCGA database and ESTIMATE algorithm, we obtained a list of genes related to TME that predicts poor prognosis in GC patients. The functions of these genes were further validated in another independent GC cohort (GEO). Our research provides a reliable way to predict the prognosis of patients with GC. Finally, further study of these genes may fully reveal the potential association between TME and GC prognosis in novel ways.

## CONFLICT OF INTEREST

The authors declare that there are no conflicts of interest.

## AUTHOR CONTRIBUTION

**Qingzhi Lan** **:** Conceptualization (lead); Data curation (equal); Formal analysis (equal); Methodology (equal); Project administration (equal); Writing‐original draft (lead); Writing‐review & editing (lead). **Peng Wang:** Conceptualization (equal); Data curation (equal); Formal analysis (equal); Methodology (equal). **Shan Tian:** Conceptualization (supporting); Data curation (supporting); Writing‐original draft (equal); Writing‐review & editing (equal). **Weiguo Dong:** Conceptualization (equal); Funding acquisition (lead); Project administration (equal); Writing‐original draft (supporting); Writing‐review & editing (supporting).

## Supporting information

Fig S1‐S2

## Data Availability

The data generated and analysed during the current study are available from the corresponding author on reasonable request. Public data and data repositories are referenced within the manuscript.
