# Supplementary material for: Mining TCGA database for genes of prognostic value in gastric cancer microenvironment
Source: J Cell Mol Med. 2020 Aug 20;24(19):11120–32. doi: 10.1111/jcmm.15595 (PMC7576220; doi:10.1111/jcmm.15595)
Supplement: Supplementary file 1 — Fig S1‐S2 [file JCMM-24-11120-s001.pdf]

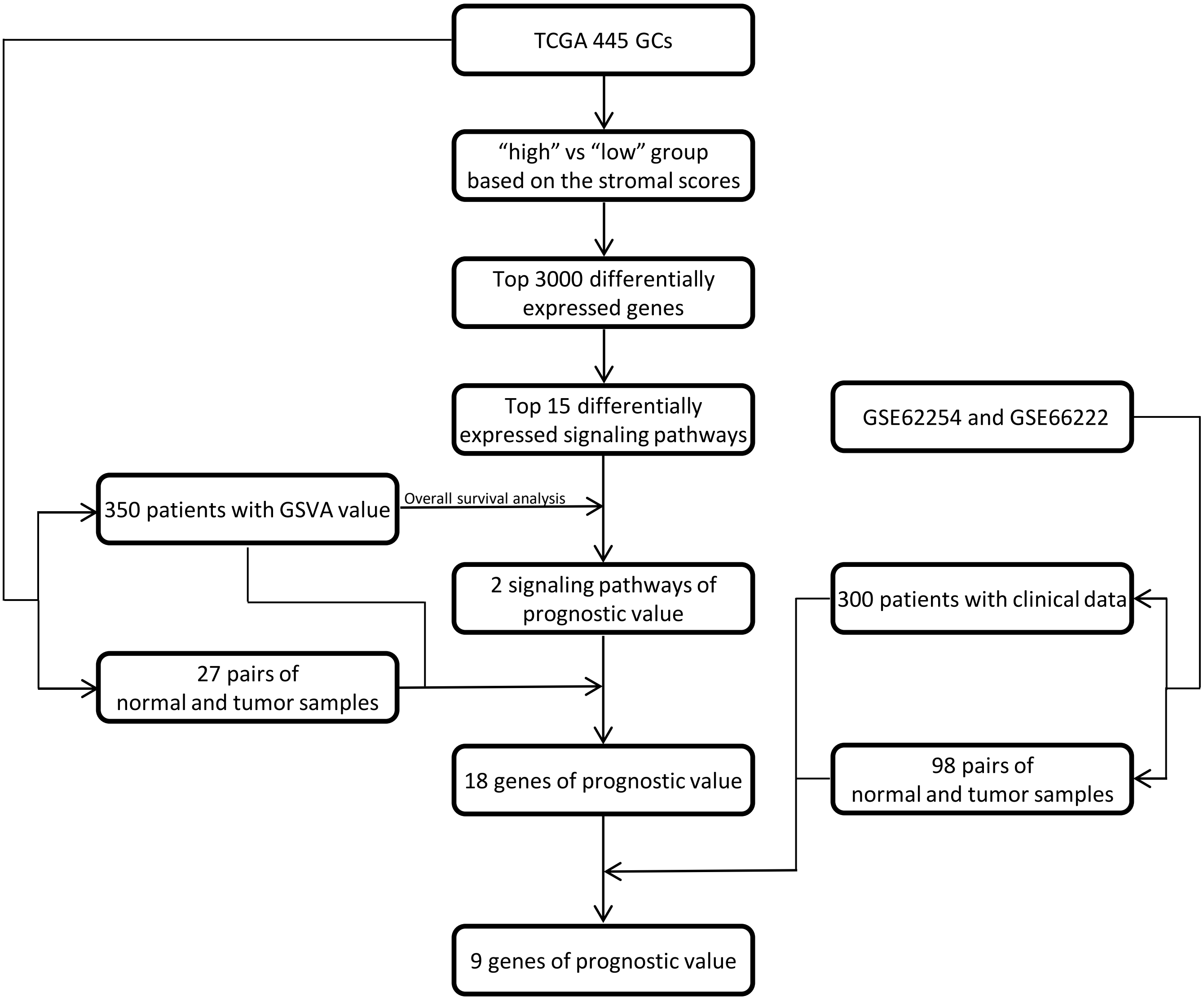

Figure 1. The flow diagram of this study.

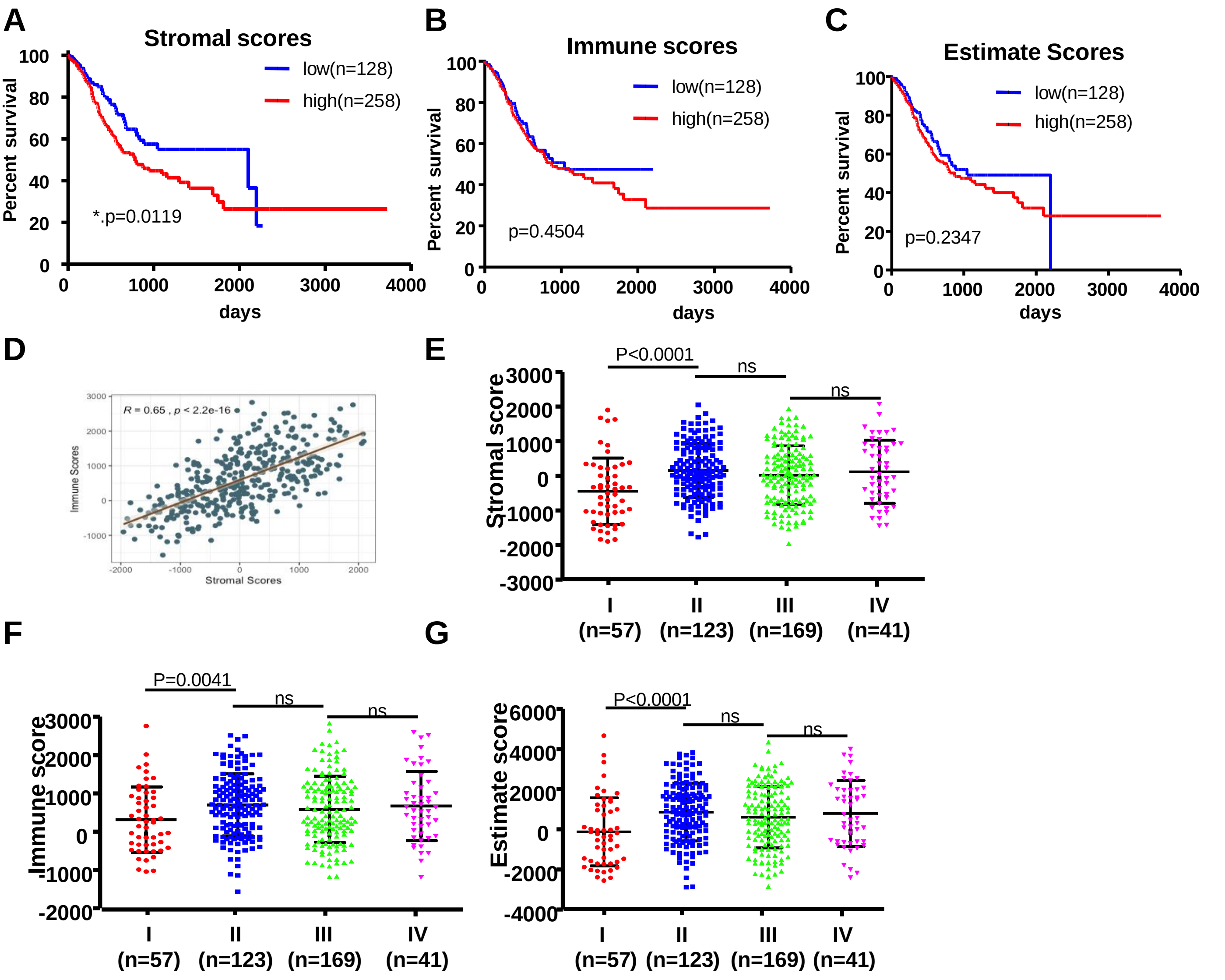

Figure 2. Stromal scores are associated with GC stages and their overall survival.

A, STAD cases were divided into two groups based on their Stromal scores: the top 2/3 of 258 cases with higher stromal scores and the bottom 1/3 of 128 cases with lower stromal scores. As shown in the Kaplan-Meier survival curve, median survival of the low score group is longer than high score group (2100 days vs. 782 days), as indicated by the log-rank test, P value is 0.0119.

B, Similarly, STAD cases were divided into two groups based on their immune scores: the 2/3 of 258 cases and the 1/3 half of 128 cases. The median survival of the low score group is longer than the high score group (1043 days vs. 869 days), however, it is not statistically different as indicated by the log-rank test P= 0.4504.

C, Similarly, STAD cases were divided into two groups based on their estimate scores: the 2/3 of 258 cases and the 1/3 half of 128 cases. The median survival of the low score group is longer than the high score group (1043 days vs. 869 days), however, it is not statistically different as indicated by the log-rank test P= 0.1688.

D, Correlation analysis of stromal-scores and immune-scores, these results show the different component of tumor microenvironment(TME) are interacted on each other.

F-G, Distribution of stromal scores, immune scores and estimate scores in the four different GC stages. Dot-plot shows that there is significant association between GC stages and the level of stromal scores, immune scores, estimate scores ,respectively (n=406, P<0.001)

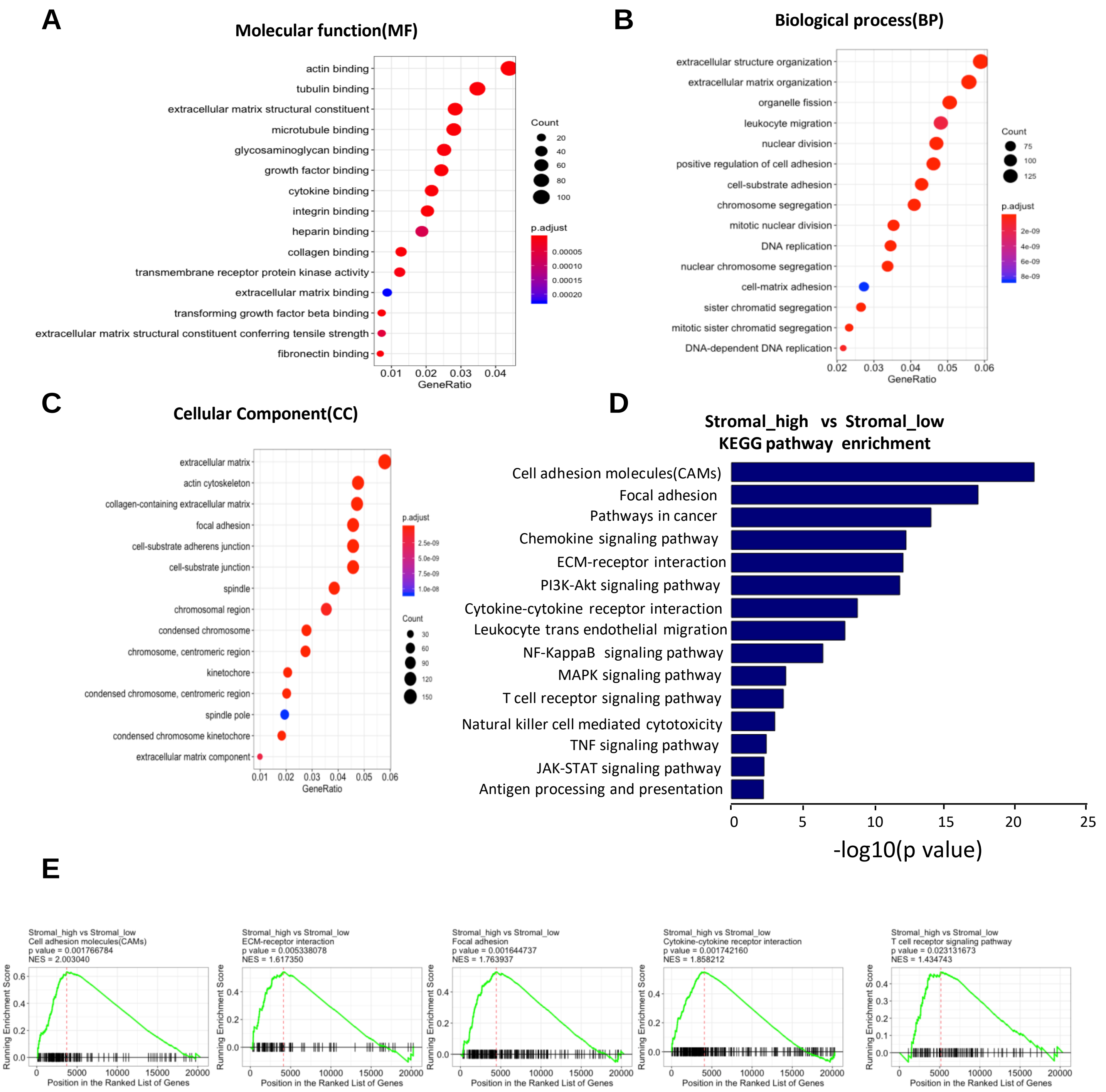

Figure 3. Comparison of gene expression profile with stromal scores in GC.

A-C, GO analysis to explore the 3000 most different genes participate in which molecular function(MF)(A), Biological process(BP)(B);Cellular component(CC)(C)

D, To explore the 3000 most different genes involved in which signaling according to the Kyoto Encyclopedia of Genes and Genomes (KEGG)datasets.

E, GSEA analysis of the data of RNA-sequencing ,in which show these KEGG signaling pathway are up-regulated in the "stromal_high" group.

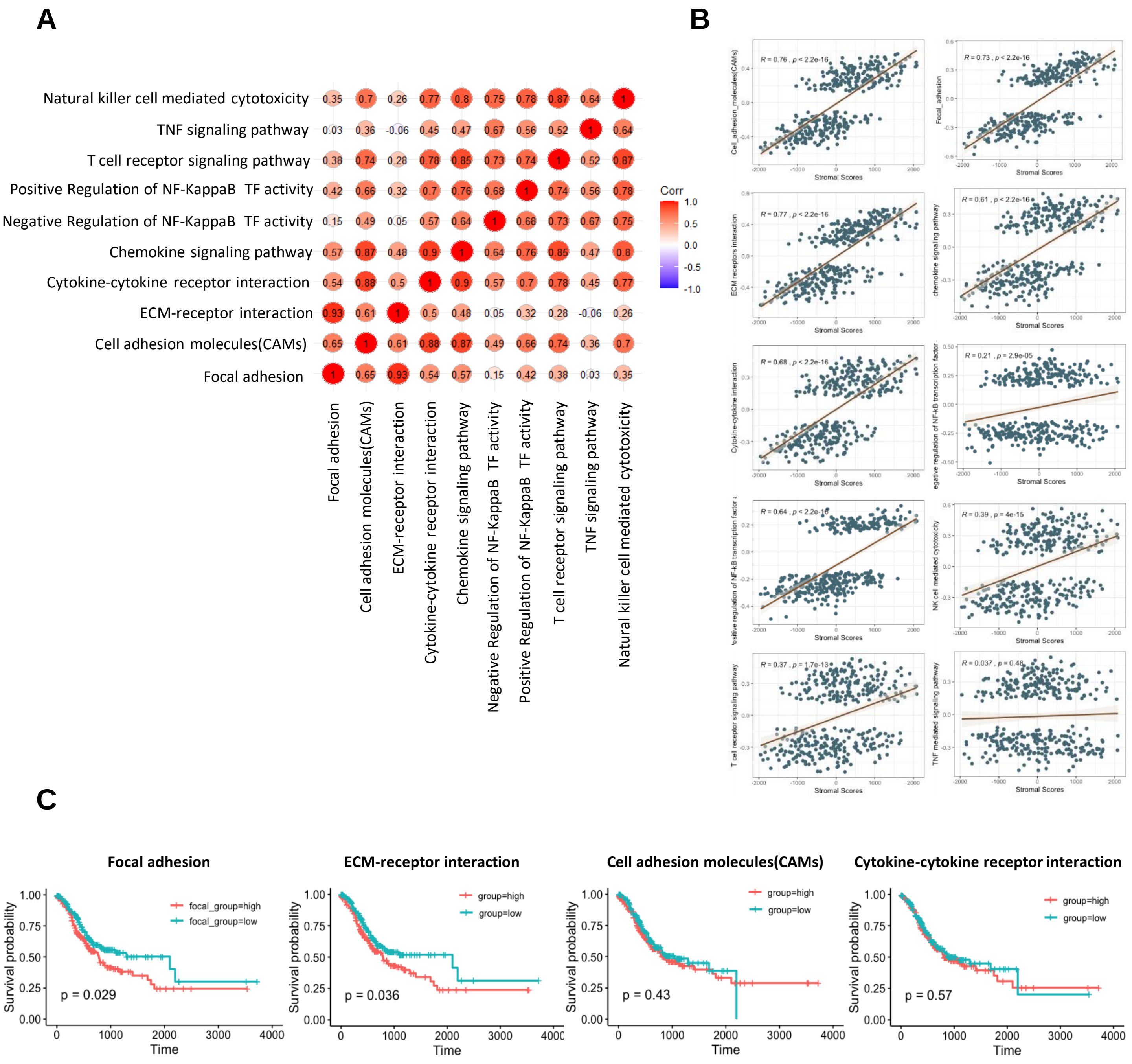

Figure 4. Correlation of expression of individual signal pathway in overall survival in TCGA.

A, Correlation of the GSVA value of the 10 signaling pathway which may take part in the poor clinical performance of the “stromal-high” group

B, Correlation between stromal scores and the GSVA values of the 10 signaling pathway ,most of which are positive correlation, apart from TNF mediated signaling pathway.

C, Survival analysis was performed on N =350 patients obtained from the TCGA cohort of gastric cancer patients that had long-term clinical follow-up data. Displayed Gene sets are downloaded from http://www.gsea-msigdb.org , most of which are downloaded from KEGG and GO datasets, GSVA scores of each signaling pathway are performed using R packages GSVA , for each signaling pathway ,the top 1/2 of 175 cases with higher GSVA scores are “high” group, and the bottom ½ of 175 cases with lower stromal scores are “low” group.

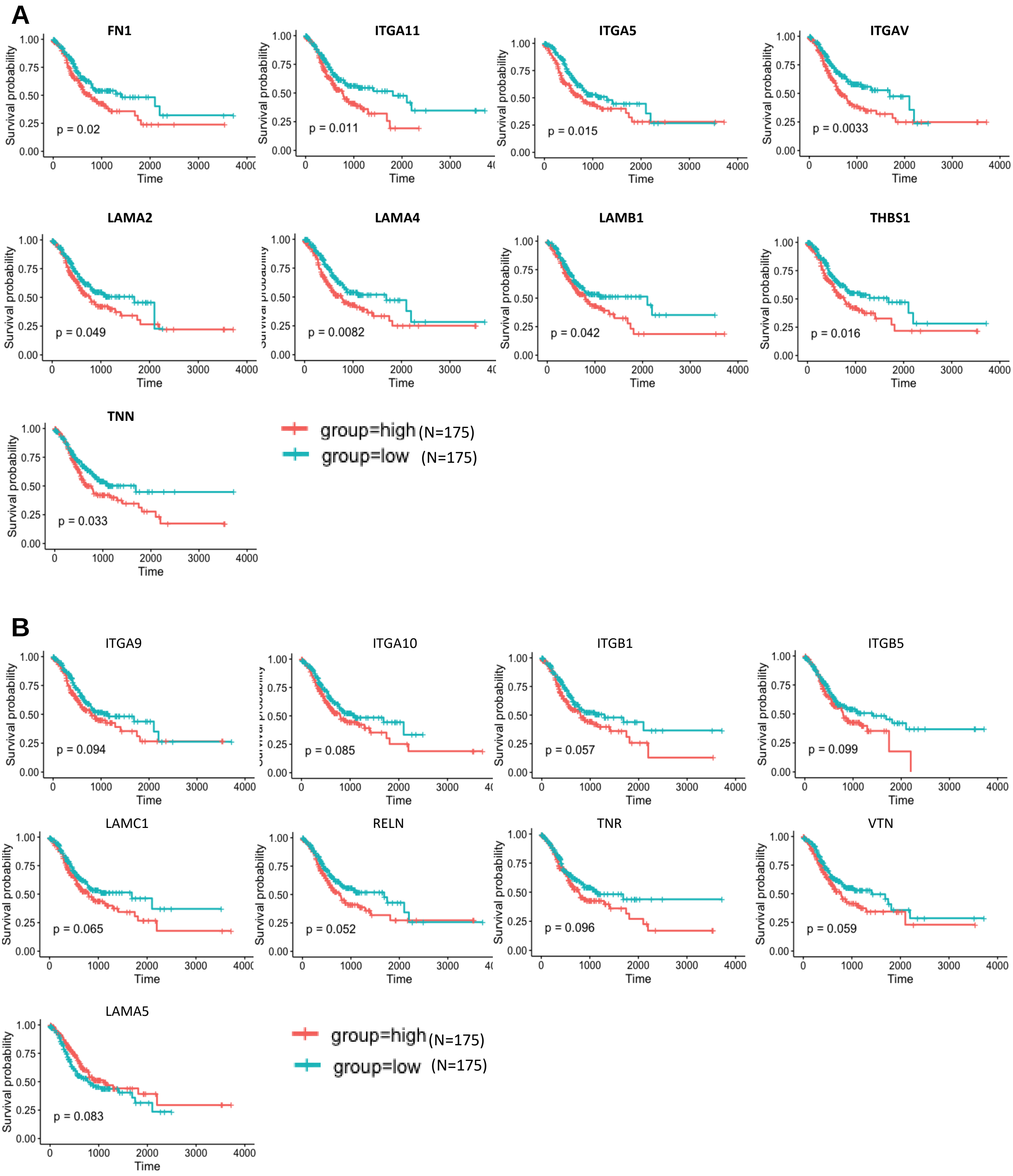

Figure 5. Correlation of expression of individual DEGs in overall survival in TCGA. Kaplan-Meier survival curves were generated for selected DEGs extracted from the comparison of groups of high (red line) and low (blue line) gene expression. P<0.05(A) or P<0.1(B) in Log-rank test. OS, overall survival in days.

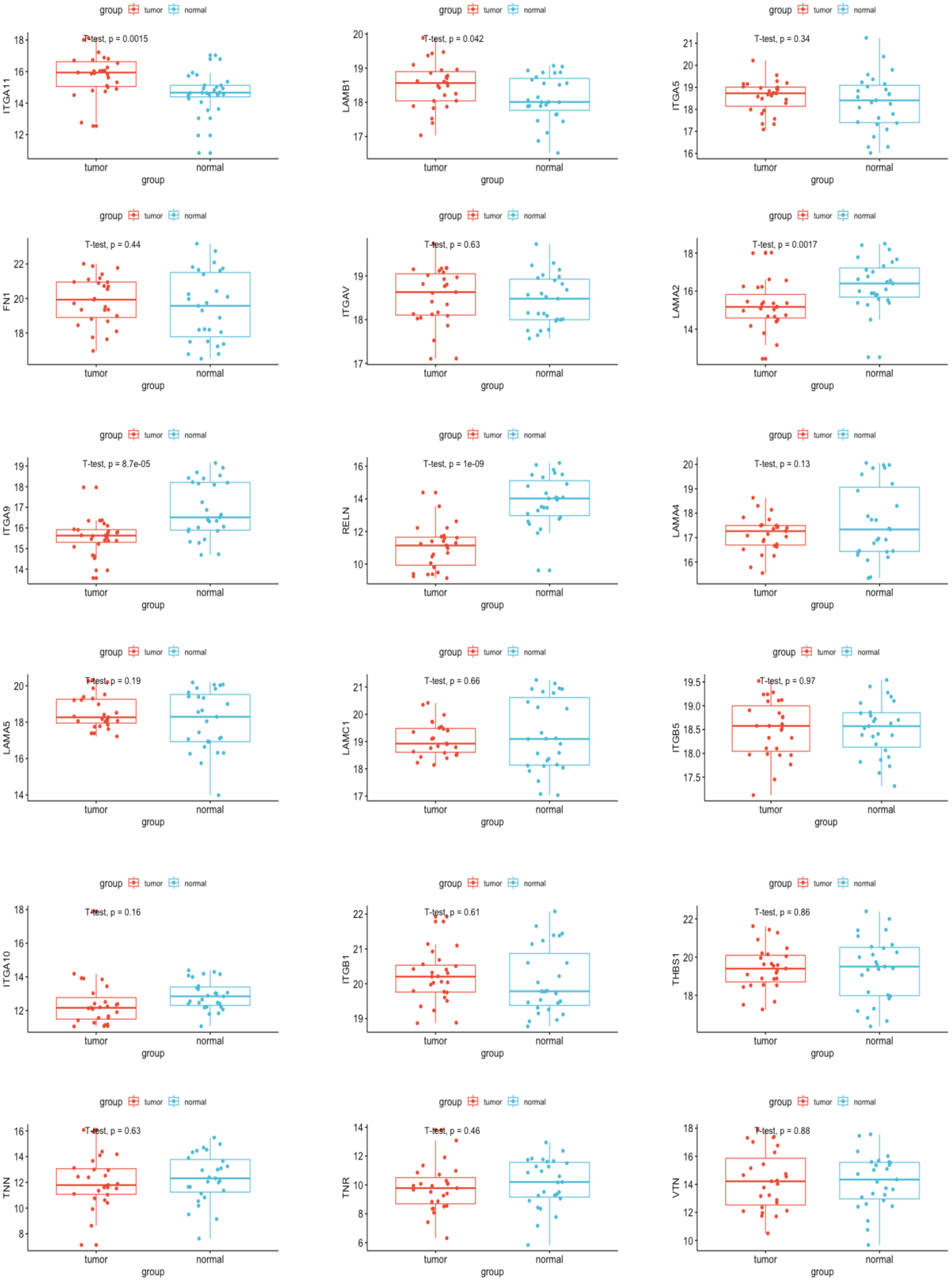

Figure 6. Different expression levels of the marker genes in TCGA cohort. Expression levels of 18 genes in figure4 in 27 pairs of tumorous samples and patient-matched normal samples in TCGA cohort.

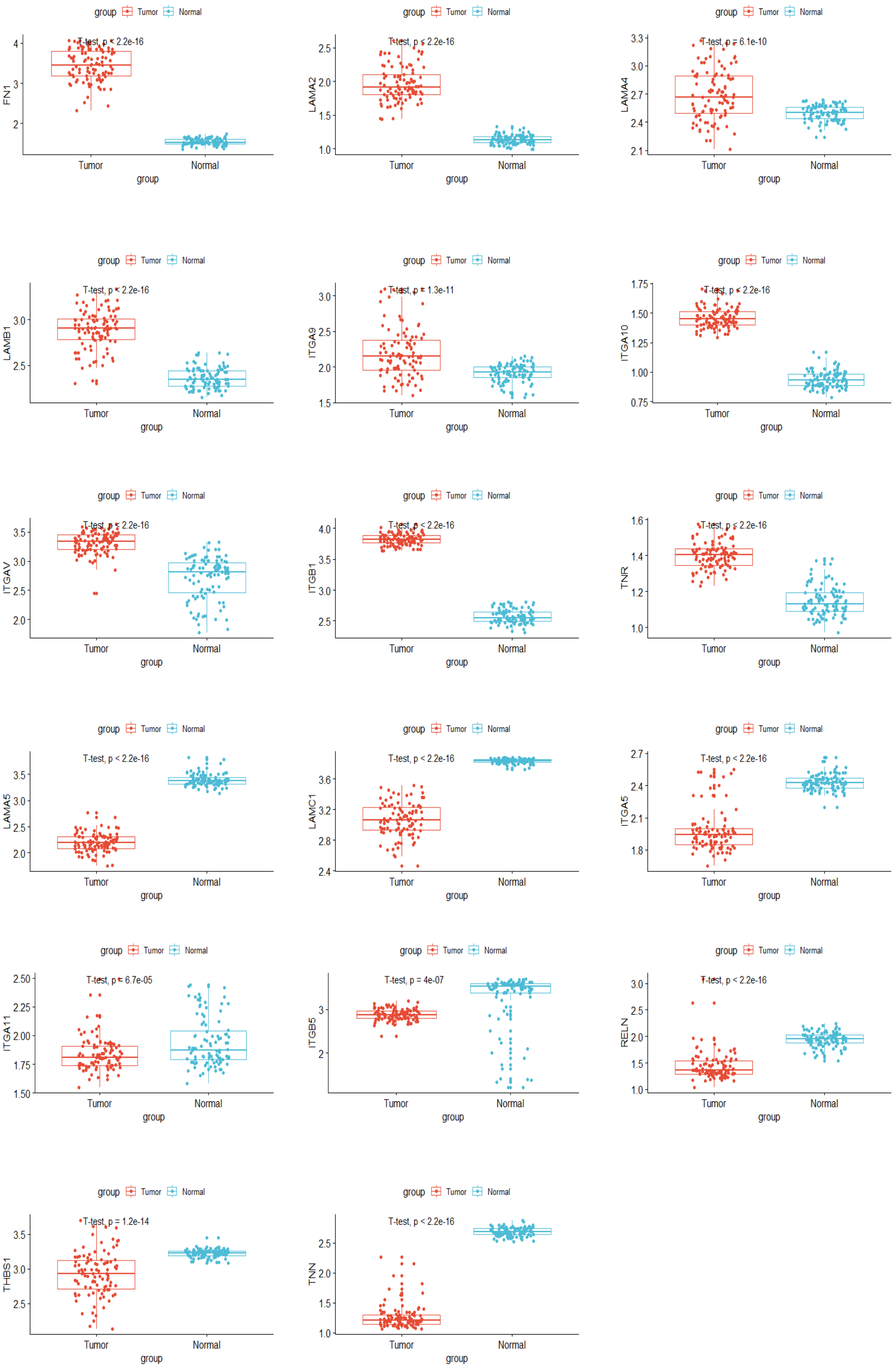

Figure 7. Validation of different expression levels of the marker genes in public datasets. Expression levels of 17 genes in 98 pairs of tumorous samples and patient-matched normal samples in GEO cohort.

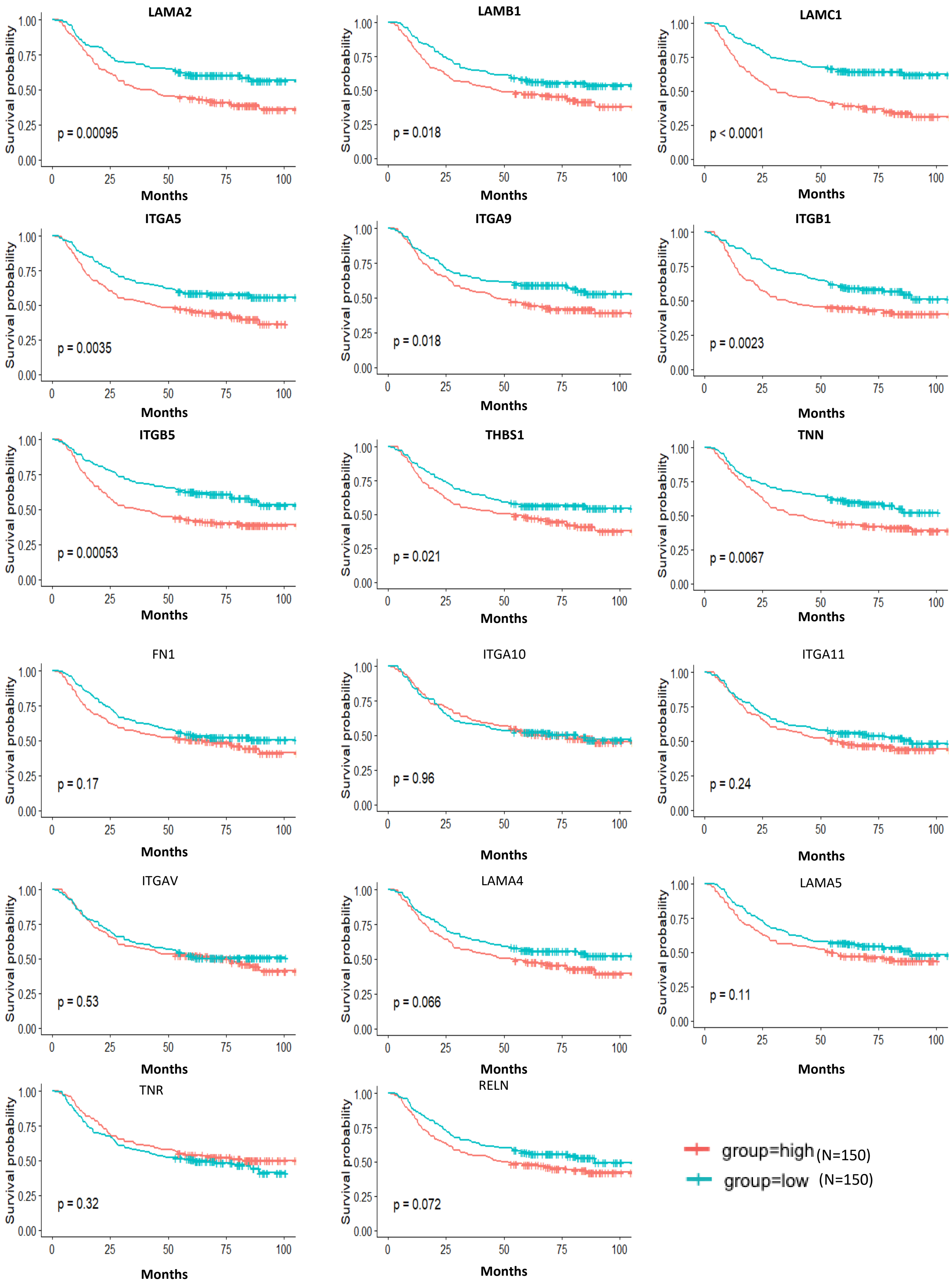

Figure 8. Validation of correlation of DEGs extracted from TCGA database with overall survival in public datasets. Kaplan-Meier survival curves were generated for selected DEGs extracted from the comparison of groups of high (red line) and low (blue line) gene expression.

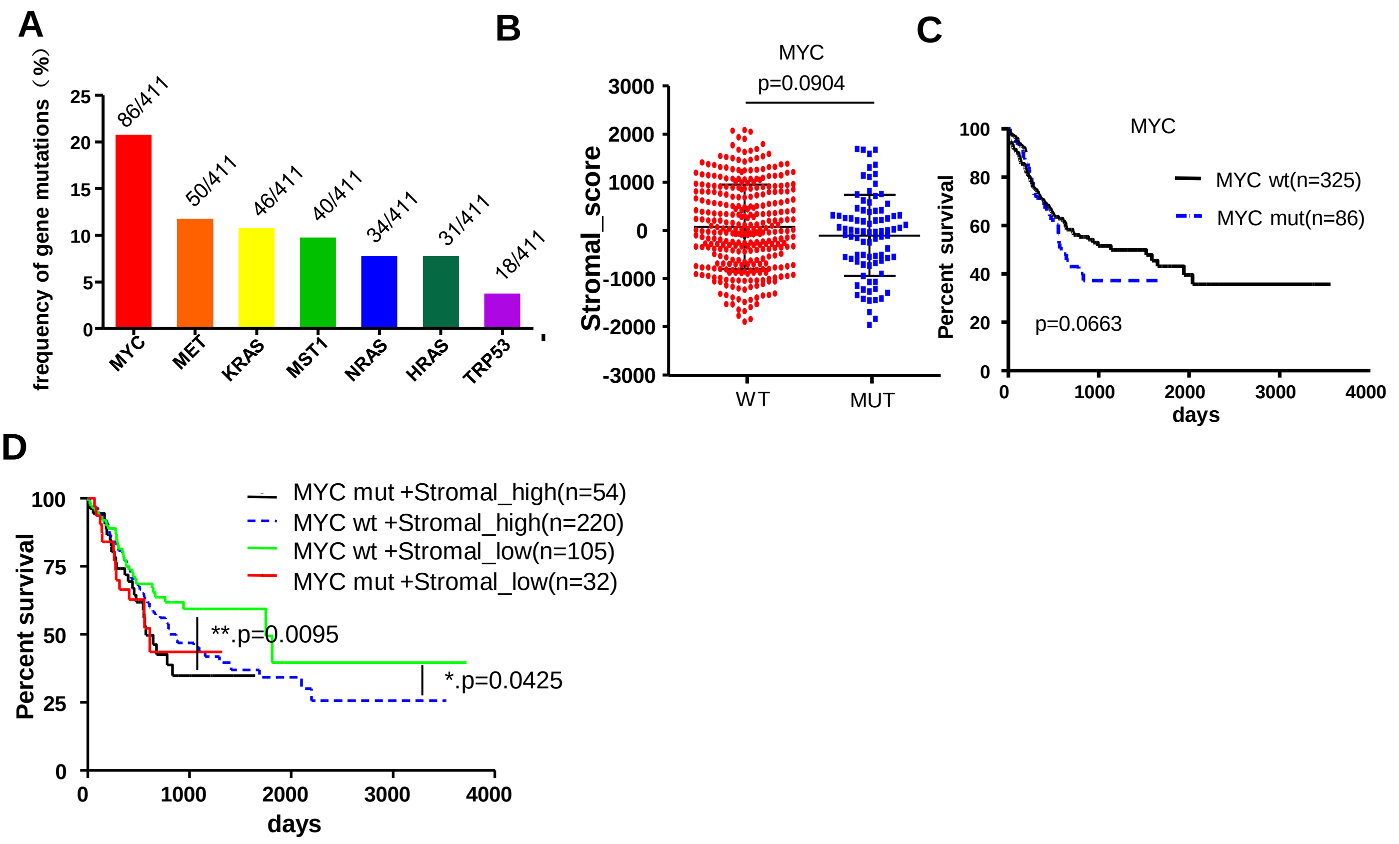

Supplementary Figure 1. MYC is the most common mutant gene in GC patients.

A, The seven most common mutational genes in GC patients in TCGA datasets and their frequency of mutations in the whole 417 GC patients, which shows myc is the most common mutant gene in GC.

B, Survival analysis of MYC-Wildtype(n=325) and MYC-mutant(n=86) in TCGA-STAD, the result indicates that myc-mutant may shorten the overall survival gastric cancer patients, although there is no significance.

C, Distribution of stromal scores in MYC-Wildtype(n=325) and MYC-mutant(n=86)patients.

D, Survival analysis of stromal scores in the context of MYC-wildtype and MYC-mutant, the results show the performance of stromal-scores on tumor progression is independent on MYC mutation, but MYC mutation can shorten the survival of the GC patients.

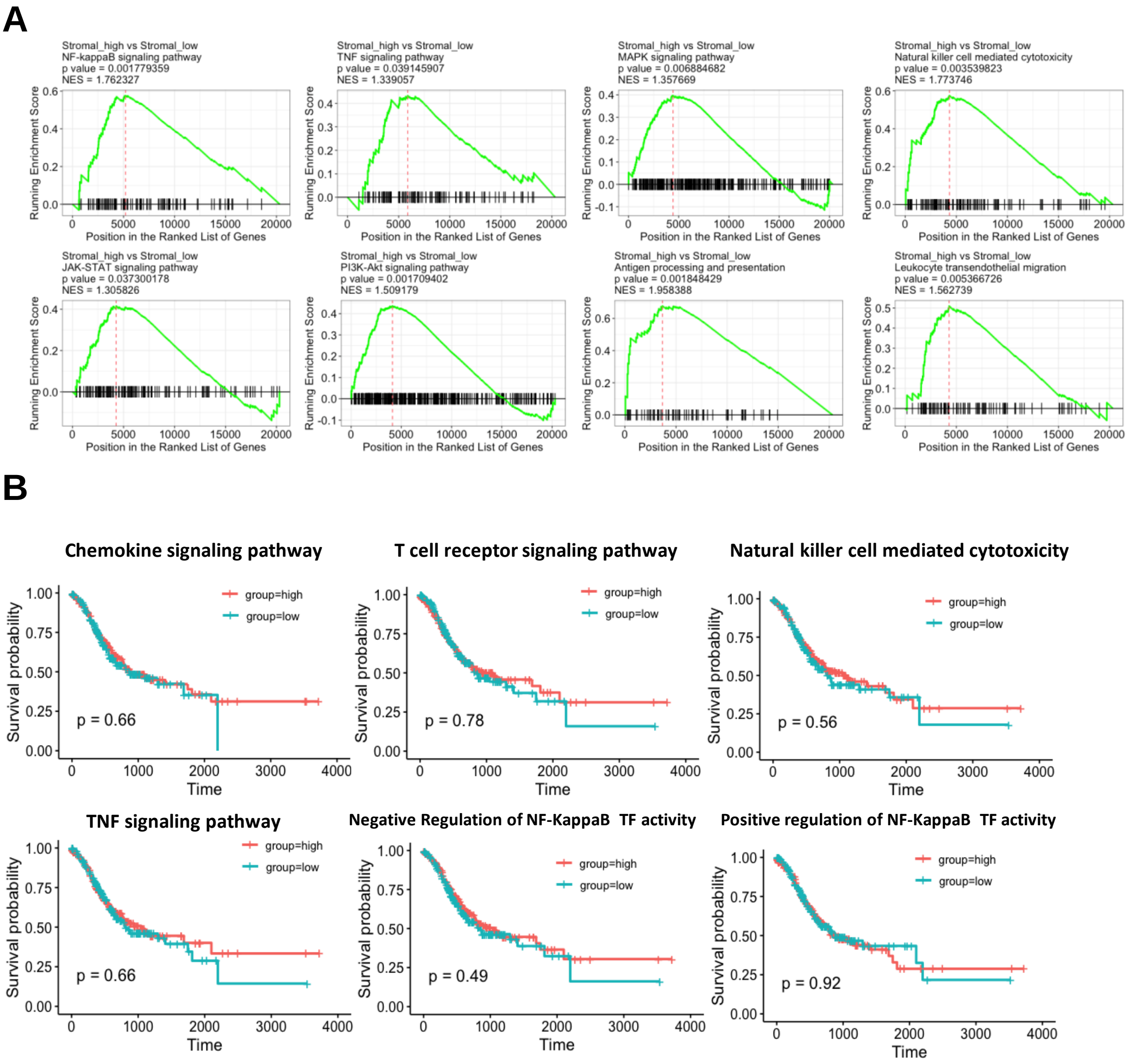

Supplementary Figure 2. Up-regulated signaling pathways in the "stromal-high" group and their effects on GC patients survival.

A, GSEA analysis of the data of RNA-sequencing ,in which show these KEGG signaling pathway are up-regulated in the "stromal-high" group.

B, Effects of signaling pathways on GC patients survival.
